# Enzyme-Assisted Extraction and Hydrolysis of Dulse (*Palmaria palmata*) Proteins: Characterisation, Antioxidant Activity and Glucose-Management Potential

**DOI:** 10.3390/foods15203717

**Published:** 2026-10-19

**Authors:** Miryam Amigo-Benavent, Yihong Chen, Anthony Temitope Idowu, Richard J. FitzGerald

**Affiliations:** 1 Proteins and Peptides Research Group, Department of Biological Sciences, Faculty of Science and Engineering, University of Limerick, V94 T9PX Limerick, Ireland; miryam.amigobenavent@ul.ie (M.A.-B.);; 2 Food, Diet and Nutrition Research Group, Health Research Institute, University of Limerick, V94 T9PX Limerick, Ireland; 3 Food Science and Technology Group, BIO3 (Pharma, Science & Engineering) Pillar, Bernal Institute, University of Limerick, V94 T9PX Limerick, Ireland

**Keywords:** bioactive peptides, carbohydrase-assisted extraction, GLP-1, INFOGEST, incretin, simulated gastrointestinal digestion, protein extraction yield

## Abstract

This study investigated the impact of temperature and enzyme-assisted extraction (EAE), specifically using carbohydrase, on protein recovery from *Palmaria palmata*. It also evaluated the antioxidant and glucose-management potential of enzymatic hydrolysates and simulated gastrointestinal digests (SGIDs) of selected protein extracts. Preincubation at 4 °C with xylanase- and cellulase-containing preparations achieved the highest protein and biomass recovery yields (75.1 ± 2.4% and 17.6 ± 0.6%, respectively). Protein extracts were hydrolysed using food-grade proteases and subsequently subjected to SGID. Both hydrolysates and SGID samples exhibited antioxidant activity, with SGID generally resulting in enhanced oxygen radical absorbance capacity (ORAC) and 2,2′-azinobis-(3-ethylbenzothiazoline-6-sulfonic acid) (ABTS) radical-scavenging activities. Neutrase^®^ hydrolysates had the highest dipeptidyl peptidase IV (DPP-IV) inhibitory activity, while similar IC_50_ values were observed within the SGID samples. All hydrolysate/SGID samples promoted insulin and active glucagon-like peptide-1 (GLP-1) secretion and inhibited DPP-IV activity in cell-based assays. These findings highlight the potential of *P. palmata* protein extract (PPPE) digests for antioxidant and glucose-management applications, supporting their potential utilisation as multifunctional food ingredients.

## 1. Introduction

The use of algae biomass in food applications is increasing in Europe [1], alongside a growing industrial interest in algal protein concentrates as sustainable alternative protein sources. *Palmaria palmata* (Linnaeus) F. Weber & D. Mohr, commonly known as dulse or dillisk, is a red seaweed (*Rhodophyta*) and one of the most widely consumed macroalgal species in Europe [2]. It is distributed along the North Atlantic coasts between 40 and 80 °N latitude [3], and is mainly harvested from wild stocks in Europe (455 tons fresh weight in 2019), with smaller quantities produced through aquaculture [1]. Pilot-scale trials have demonstrated successful cultivation of *P. palmata* in land-based tank systems and at sea, supporting the sustainable production of its biomass [4].

*P. palmata* protein content is reported to range between 7 and 19% (*w*/*w*) dry weight (dw) [3] when using a nitrogen-to-protein conversion factor of 4.7 [5]. This protein content is higher than that reported for other seaweed species, making *P. palmata* a promising alternative protein source for human consumption [6,7]. It is well documented that the protein content and composition of *P. palmata* varies according to season, harvest location and cultivation conditions [8,9]. Laboratory-scale aquaculture studies, with potential for scale-up, have demonstrated that protein content can be enhanced through manipulation of cultivation parameters, including culture medium composition, photoperiod and lighting colour conditions [10,11,12]. Additionally, *P palmata* proteins contain a balanced profile of essential and non-essential amino acids [5,7,13].

The extraction of proteins from *P. palmata*, similar to other macroalgal species, remains challenging because proteins are entrapped within densely packed cortical cells [14], limiting extraction efficiency and industrial applicability. A range of approaches including alkaline extraction, EAE (using carbohydrases and proteinases), and deep eutectic solvents (DESs), as well as physical techniques such as ultrasound and pulsed electric field treatment, have been investigated either individually or in combination [6,13,15,16,17,18,19,20,21] in order to enhance protein recovery. Protein recovery varies considerably depending on the extraction approach. The highest recoveries have been reported for EAE combined with alkaline extraction (70%) [13] and alkaline extraction alone (45–61%) [15,16], whereas lower recovery values have been obtained using DESs (14.4%) [14], microwave-assisted extraction (47.6%) or ultrasound-assisted extraction (6.9–12.2%) [21]. However, many studies do not report key performance indicators such as protein content, protein extraction yield and biomass extraction yield. Details on these parameters would facilitate comparisons among extraction strategies and provide valuable information regarding their scalability and industrial feasibility. Furthermore, the effect of extraction temperature during EAE appears to have received limited attention and has not been systematically evaluated.

Given the growing interest in the use of *P. palmata* proteins as food ingredients, extraction processes need to be developed under food-grade conditions while taking the composition of the *P. palmata* cell wall into account. Its cell wall predominantly consists of β-(1→3)/β-(1→4)-D-xylans, together with smaller amounts of cellulose and β-(1→4)-D-xylans [22]. Consequently, carbohydrate preparations containing xylanases and cellulases [13,15,18] may be particularly suitable for EAE. In contrast, while protease-mediated EAE can improve nitrogen recovery, its usage leads to protein breakdown, potentially altering both the technofunctional [23] and the sensory attributes of the resulting ingredients [24].

In addition to maximising protein recovery, preservation of intact protein structure may be particularly important when extracts are intended for use as technofunctional food ingredients or for the targeted generation of specific bioactive peptide sequences with health-promoting properties. In addition to its nutritional value, the proteins from *P. palmata* and its associated peptides in *P. palmata* protein hydrolysates (PPPHs) have demonstrated glucose-management, cardioprotective and antioxidant activities during *in vitro* [25,26,27,28,29,30,31,32], *in silico* [33], and *in vivo* studies [34,35,36]. Consequently, PPPHs show considerable potential as biofunctional food ingredients targeted at the prevention and management of various cardiometabolic conditions. However, limited information is available regarding the susceptibility of carbohydrase EAE-derived proteins to proteolysis and gastrointestinal digestion, as well as the effects of the resulting peptides on glucose-management mechanisms and antioxidant activity *in vitro* and *ex situ*.

Therefore, the aims of this study were to investigate the impact of incubation temperature during carbohydrase treatment combined with alkaline extraction on the recovery of *P. palmata* proteins, to report key extraction parameters, to generate protein hydrolysates and their SGIDs, and to evaluate their glucose-management and antioxidant activities using *in vitro* assays and cell-based models.

## 2. Materials and Methods

### 2.1. Materials

Five different commercially available food-grade carbohydrase preparations were used in this study. DEPOL^TM^ 692L (800 cellulase U/g, 535 pectinase U/g), DEPOL^TM^ 761P (xylanase 800 U/g), DEPOL^TM^ 793L (5500 beta glucanase U/g, 5000 pectin lyase U/g, 1200 cellulase U/g), CELLULASE 13L (1500 cellulase U/g) and the protease PROMOD^TM^ 439L (220 casein protease U/g) were kindly provided by Biocatalysts (Wales, UK), whereas EXTRALYVE LA2X (1200 cellulase U/g, 3000 xylanase U/g, 3000 polygalacturonase U/g) was kindly provided by Soufflet Biotechnologie (Normandie, France). The activities presented herein correspond to those provided in the suppliers’ technical information at optimal temperatures (30–65 °C).

The food-grade commercial protease preparations Alcalase^®^ 2.4 L (≥2.4 U/g, product P4860), Neutrase^®^ 0.8 L (≥0.8 U/g, product P1236), Flavourzyme^®^ (≥500 U/g, product P6110) and Bromelain from pineapple stem (3–7 U/mg protein, product B4882) were obtained from Sigma-Aldrich (Wicklow, Ireland). The endo-cellulase (CellG5 method, EP 2740800 AI) and endo-xylanase (XylX6 method) activity assay kits were purchased from Megazyme (Wicklow, Ireland). The Pierce™ BCA protein assay kit and PrestoBlue™ Cell Viability Reagent were purchased from ThermoFisher Scientific (Dublin, Ireland). BRIN-BD11 cells (passage numbers 18–24) were kindly provided by Dr Peter Flatt from Ulster University (Belfast, UK). STC-1 cells (2333 ATCC CRL3254, passage numbers 23–35) and HepG2 cells (passage number 100–114) were purchased from the American Tissue Culture Collection (Manassas, VA, USA). Human Caucasian colon adenocarcinoma (Caco-2) cells (passage numbers 50–57), Minimum Essential Medium Eagle (MEM), Roswell Park Memorial Institute-1640 (RPMI-1640), Dulbecco’s Modified Eagle Medium (DMEM), foetal bovine serum (FBS), and penicillin–streptomycin were purchased from Merck (Wicklow, Ireland). Syringe filters (0.2 μm, Filtropur S) were supplied by Carl Stuart Ltd. (Dublin, Ireland). Glucagon-like peptide-1 (Active) ELISA (MILLIPLEX Mouse Metabolic Hormone Expanded Panel—Metabolism Multiplex Assay EGLP-35K) and 2,2′-azobis(2-amidinopropane) dihydrochloride (ABAP) were provided by Merck (Wicklow, Ireland). The ultrasensitive insulin ELISA kit was obtained from Crystal Chem (Itasca, lL, USA). *P. palmata* biomass was kindly provided by Pure Ocean Algae Ltd. (Cork, Ireland) and Irish Seaweed Consultancy Ltd. (ISC, Galway, Ireland). All other reagents were of analytical grade.

### 2.2. Seaweed Collection and Preparation

Wild *P. palmata* biomass collected between February and August 2020 from the West Coast in Ireland by ISC (Galway, Ireland) was used for the protein extraction method development studies. After collection, the samples were washed with sterilised seawater and freeze-dried. The freeze-dried biomass was milled using a blend mixer (Krups F203 Grinder, Millville, NJ, USA), sieved (ca. 3 mm mesh size) and pooled, resulting in a sample with a protein content of 10.86% (*w*/*w*, protein content). The pooled sample was stored in an airtight container for subsequent protein extraction experiments.

Tank-cultured *P. palmata* biomass (kindly provided by PureOcean Algae Ltd. (West Cork, Ireland with the support of ISC) was used for the generation of protein hydrolysates and the associated *in vitro* and *in situ* bioactivity assays. After collection, the samples were washed with sterilised seawater, freeze-dried, pooled (16.13%, *w*/*w* protein content), milled, sieved (ca. 3 mm mesh size) and stored in an airtight container for subsequent experiments.

### 2.3. Protein Extraction Method Development

**Figure 1:**
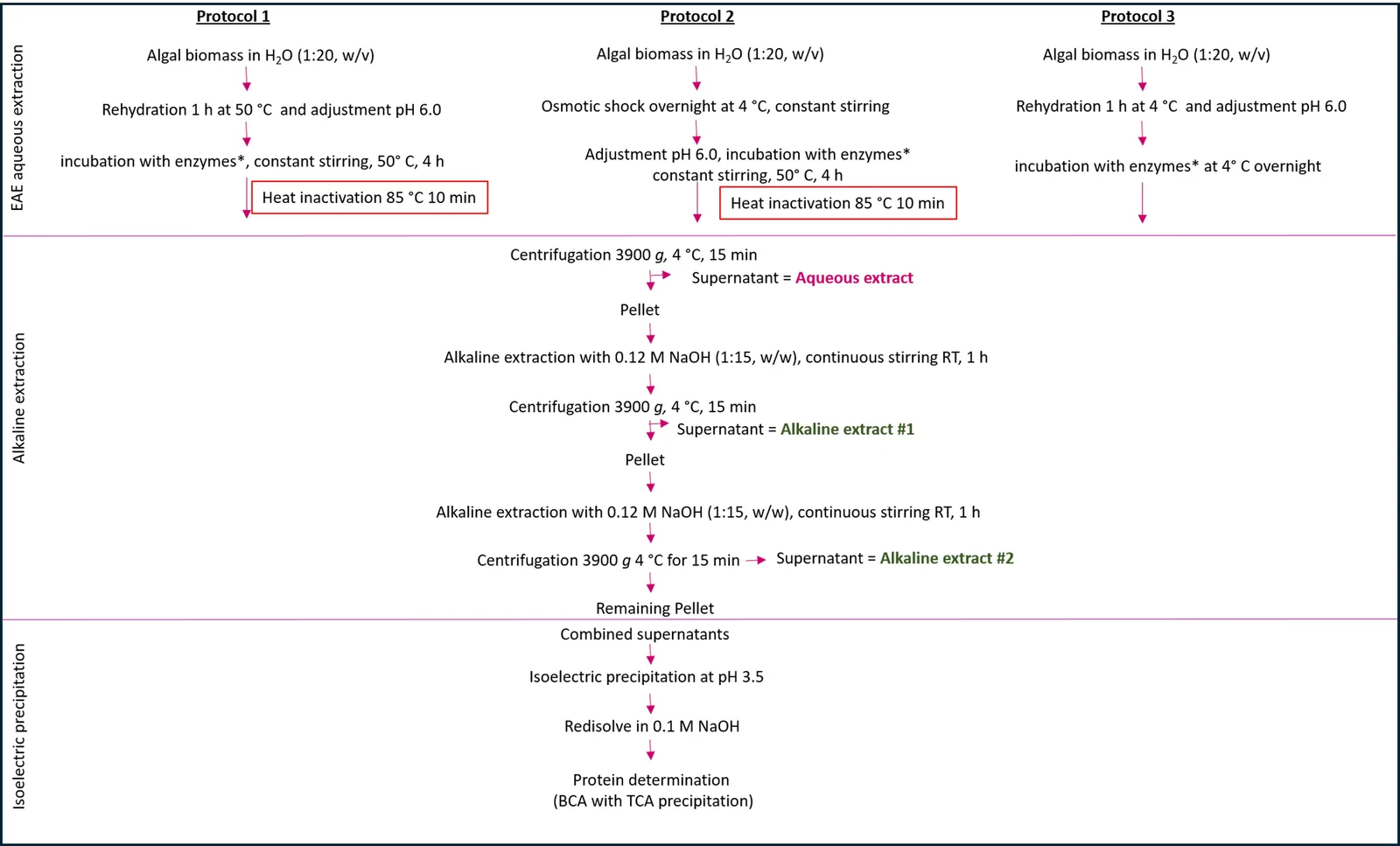
Details of the different protocols employed during the carbohydrase enzyme-assisted extraction (EAE) of *Palmaria palmata* proteins. Different incubation times and temperatures were studied during the aqueous extraction phase with the inclusion of carbohydrases, whereas the alkaline extraction and isoelectric precipitation steps were carried out under similar conditions. Protocol 3 was also performed without heat inactivation. RT: room temperature, BCA: bicinchoninic acid, TCA: trichloroacetic acid. * indicates the inclusion of CHOase(s); see the Material and Methods Section for further details.

The aqueous and alkaline extraction method described by Harnedy and FitzGerald [15] and Idowu et al. [13] was modified to include EAE with carbohydrases (CHOases) under different incubation temperatures and durations (Figure 1).

Three different protein extraction parameters/protocols were tested, involving changes to the aqueous extraction step while also including EAE (Figure 1). In all cases, 1 g of pooled *P. palmata* biomass was initially placed in a 50 mL conical-bottomed plastic container with 18 mL of distilled water. In protocol 1 (see Figure 1), the biomass was left to hydrate at 50 °C for 1 h, and the pH was then adjusted to pH 6.0 using 2 M HCl, the CHOases were then added and the volume was made up to 20 mL. The reaction was incubated at 50 °C for 4 h followed by heat inactivation of the enzyme(s) by heating at 85 °C for 10 min in a water bath. In protocol 2 (Figure 1), the EAE was carried out after overnight osmotic shock of the biomass in distilled water at 4 °C, after which the pH was adjusted to pH 6.0, the CHOases were then added, and the volume was made up to 20 mL. The reactions were then incubated with gentle stirring at 50 °C for 4 h followed by heat inactivation at 85 °C for 10 min. In protocol 3 (Figure 1), the biomass was allowed to hydrate at 4 °C for 1 h, the pH was then adjusted to pH 6.0, the CHOases were added, the volume was made up to 20 mL and the mixture was incubated with constant gentle stirring at 4 °C overnight. Liquid enzyme preparations were added on a volume-to-biomass weight basis (% *v*/*w*), whereas powdered enzyme preparations were added on a weight-to-biomass weight basis (% *w*/*w*). EAE was carried out at enzyme-to-substrate ratios (E:S, biomass weight basis) of 0.01% (*v*/*w*) for DEPOL^TM^ 692L, 0.02% (*w*/*w*) for DEPOL^TM^ 761P, 0.001% (*v*/*w*) for DEPOL^TM^ 793L and 0.05% (*v*/*w*) for CELLULASE 13L, following the suppliers’ recommendations. A control without CHOase(s) was also performed in order to compare EAE vs. direct aqueous and alkaline extraction per se.

Following aqueous incubation with the CHOase(s), alkaline extraction was performed using the steps described in Figure 1. Briefly, the mixture was initially centrifuged at 3900× *g* at 4 °C for 15 min, where the supernatant obtained was termed the aqueous extract. The pellet obtained was resuspended with 15 mL of 0.12 M NaOH and it was then gently stirred at room temperature for 1 h. A second centrifugation step was carried out at 3900× *g* and 4 °C for 15 min, where the supernatant obtained was termed alkaline extract #1. This step was repeated to obtain alkaline extract #2. All the supernatants (aqueous, alkaline #1 and #2) were combined and the pH was adjusted to pH 3.5; the samples were left to stand for 30 min and were then centrifuged at 3900× *g* at 4 °C for 30 min. A pH value of 3.5 was selected for protein precipitation following preliminary experiments conducted at pH 2.0, 2.5, 3.0, 3.5, 4.0 and 4.5 (Figure S1) which showed that pH 3.5 led to optimal precipitation of the *P. palmata* proteins. The pellet obtained after pH 3.5 treatment contained the protein-enriched fraction. All extractions were carried out in independent triplicates.

The protein content in the extracts was determined using a modification of the PierceTM BCA assay involving initial protein precipitation with trichloroacetic acid (TCA). Briefly, the protein-enriched pellet obtained following protocols 1–3 was resuspended in 5 mL of distilled water and the pH was adjusted to pH 11.0 using 0.1 M NaOH to solubilise all the proteins; the final volume was then adjusted to 6 mL. An aliquot (500 uL) of the solubilised proteins was transferred to a 1.5 mL plastic vial into which 500 uL of distilled water, 100 uL of 0.15% (*w*/*v*) sodium deoxycholate and 100 uL of 72% (*w*/*v*) TCA were added, mixed and left to stand on ice for 15 min to precipitate the protein. After the precipitation, the vials were centrifuged at 20,817× *g* at 4 °C for 15 min. The supernatant was discarded, and the pellet was resuspended in 1 mL of 0.1 M NaOH. A calibration curve using bovine serum albumin dissolved in 0.1 M NaOH (20–250 μg/mL) was prepared and the protein therein was precipitated and resuspended in the same manner as described above. A 25 uL aliquot of the protein solution was added into a 96-microwell plate along with 200 μL of BCA working solution. The absorbance at 570 nm was read following incubation for 30 min in the dark. The concentration of protein in the test samples was calculated by interpolating from the calibration curve. These experiments allowed establishment of the optimum incubation temperature and duration conditions for the subsequent experiments.

### 2.4. Carbohydrase EAE for P. palmata Protein Extraction

As protocol 3 provided the highest protein recovery (without heat inactivation), this protocol was repeated with DEPOL^TM^ 692L, DEPOL^TM^ 761P, DEPOL^TM^ 793L, CELLULASE 13L and EXTRALYVE LA2X individually and with DEPOL^TM^ 692L, DEPOL^TM^ 793L, CELLULASE 13L in combination with DEPOL^TM^ 761P (to include xylanase and cellulase activity). The obtained PPPEs were freeze-dried and their protein content was determined using the micro-Kjeldahl method as previously described [13], applying a nitrogen-to-protein conversion factor of 4.7 [5]. Protein recovery yield was calculated based on the protein content in the initial biomass and the protein content of the recovered PPPE, according to Equation (1):

(1) $$\mathrm{Protein}\;\mathrm{recovery}\;\mathrm{yield}\;\left(\%\right)=\frac{\mathrm{Protein}\;\mathrm{content}\;\mathrm{in}\;\mathrm{the}\;\mathrm{PPPE}\times 100}{\mathrm{Protein}\;\mathrm{content}\;\mathrm{in}\;\mathrm{initial}\;\mathrm{biomass}}$$

Biomass extraction yield was determined gravimetrically after freeze-drying and calculated relative to the initial biomass weight according to Equation (2):

(2) $$\mathrm{Biomass}\;\mathrm{recovery}\;\mathrm{yield}\;\left(\%\right)=\frac{\mathrm{Amount}\;\mathrm{of}\;\mathrm{extracted}\;\mathrm{biomass}\;\mathrm{in}\;\mathrm{the}\;\mathrm{PPPE}\left(g\right)\;\times \;100}{\mathrm{Amount}\;\mathrm{of}\;\mathrm{initial}\;\mathrm{biomass}\;(g)}$$

### 2.5. Enzymatic Hydrolysis and In Vitro Gastrointestinal Digestion of PPPE

The carbohydrase EAE PPPE obtained with a combination of DEPOL^TM^ 761P and CELULASE 13L^®^ in Section 2.4 was enzymatically hydrolysed using food-grade proteases yielding PPPHs. The hydrolysis reaction was carried out at 10% (*w*/*v*) total solids (TS), with PPPE allowed to hydrate while gently stirring at 50 °C for 1 h. The pH of the suspension was adjusted to pH 7.0 using 2 M NaOH. PPPHs were generated using Alcalase^®^ alone or in combination with Flavourzyme^®^ or Promod^TM^ 439L or individually with Neutrase^®^ or Bromelain. Hydrolysis was carried out at an enzyme-to-substrate ratio (E:S) of 1% (*v*/*w*) at 50 °C for 4 h with constant stirring under free-fall pH conditions. The hydrolysates obtained were heat-inactivated at 85 °C for 10 min and then freeze-dried.

A portion of these hydrolysates or PPPEs (control) were subjected to *in vitro* simulated gastrointestinal digestion (SGID) following the INFOGEST 2.0 consensus protocol [37] prior to assessing their bioactivity using *in vitro* and cell culture (*in situ*) methods with some modifications. Briefly, 0.70 g (55.16%, *w*/*w*, protein content) of freeze-dried PPPH or PPPE was dissolved in water and salivary fluid to perform oral digestion with human alpha-amylase (final concentration 75 U/mL in the final mixture) with constant stirring (150 rpm) at 37 °C for 2 min. The final volume of the oral phase was diluted 1:1 (*v*/*v*) with simulated gastric fluid containing pepsin from porcine gastric mucosa (final pepsin concentration 2000 U/mL in the final mixture). The gastric digestion was incubated with constant stirring (150 rpm) at 37 °C for 120 min. The intestinal digestion was performed by adding Pancreatin from porcine pancreas (100 U trypsin activity/mL in the final mixture). Bile salts were not included during these digestions to avoid cytotoxic effects during the cell-based experiments. The digestion was stopped by heat inactivation at 85 °C for 15 min in a water bath. The samples were then freeze-dried. The protein equivalent content in the SGID samples was calculated considering only the protein from *P. palmata*, and no losses took place during the digestion. The tubes used for the digestion were pre-weighted before the digestion and after freeze-drying to calculate the equivalent amount of original protein per unit weight of the starting freeze-dried powder (fdp).

The degree of hydrolysis (DH), and the molecular mass distribution analyses were carried out as described previously [38]. A control unhydrolysated sample was also prepared using the same conditions as those used for the hydrolysates but without the addition of enzyme; this control was also used for the SGID samples. All samples were freeze-dried prior to further analysis.

### 2.6. In Vitro Antioxidant Activity (ORAC and ABTS)

The antioxidant activity of the PPPE, its hydrolysates (PPPHs) and the SGID samples was determined using the oxygen radical absorbance capacity (ORAC) and the ABTS decolourisation assays as described previously [39]. For both assays the samples were prepared at 20 mg fdp/mL and diluted to the appropriate dilution. The analysis was carried out in triplicate. The results were expressed as μmol of trolox equivalents (TE)/g fdp.

### 2.7. In Vitro Dipeptidyl Peptidase IV (DPP-IV) Inhibitory Activity

The DPP-IV inhibitory activity of the PPPE, PPPH and SGID samples was determined as described previously [39]. The samples were prepared at 50 mg fdp/mL and serially diluted in order to estimate the % of inhibition at different concentrations. The analysis was carried out in triplicate. The results were expressed as the concentration required to inhibit 50% of enzyme activity, reported as the half-maximal inhibitory concentration IC_50_ (mg fdp/mL).

### 2.8. Cell-Based Glucose Regulatory Assessment

The insulin secretion promotion potential of PPPE, its hydrolysates and their SGID samples was determined using rat clonal pancreatic BRIN-BD11 β-cells as described previously by Harnedy et al. [40]. *P. palmata* samples were rehydrated at 10 mg protein equivalent/mL in Krebs-Ringer bicarbonate buffer containing 1.1 mM of glucose at 4 °C for 18 h. The solutions were centrifugated at 2580× *g* for 5 min, then sterilised by filtration using 0.2 µm syringe filters. The supernatants were collected and used for cytotoxicity and insulin secretion assays. Cytotoxicity was tested at concentrations of 1.0 and 2.5 mg protein equivalent/mL following 2 h of treatment using PrestoBlue™. The promotion of insulin secretion was tested in the supernatants, after incubation with the samples at a concentration of 2.5 mg protein equivalent/mL for at 37 °C for 20 min, using a Mercodia Ultrasensitive Rat Insulin ELISA kit (Mercodia, Uppsala, Sweden) following the manufacturer’s instructions. All analyses were performed in triplicate. Insulin secretion was expressed as ng of insulin/mg cellular protein. Negative controls with Krebs-Ringer buffer containing 1.1 mM glucose and a positive control with 10 mM L-alanine dissolved in Krebs-Ringer buffer were also included during the assay.

The stimulation of active GLP-1 secretion by PPPE, PPPHs and their SGID samples was determined using murine intestinal enteroendocrine STC-1 cells as described previously [41]. *P. palmata* samples were rehydrated at 10 mg protein equivalent/mL in Krebs-Ringer bicarbonate buffer containing 1.8 mg/L of glucose at 4 °C for 18 h. The solutions were centrifugated at 2580× *g* for 5 min, then sterilised by filtration using 0.2 µm syringe filters. The supernatants were collected and used for cytotoxicity and insulin secretion assays. Cytotoxicity was tested at concentrations of 1.0 and 2.5 mg protein equivalent/mL following 4 h of treatment using PrestoBlue™. STC-1 cells were incubated with the samples at 2.5 mg protein equivalent/mL at 37 °C for 4 h. After the incubation period, 5 µL of 100X Halt Protease and Phosphatase Inhibitor was added to the wells to inactivate endogenous DPP-IV activity and to preserve active GLP-1. Active GLP-1 was quantified using the Glucagon-Like Peptide-1 (Active) ELISA Kit 96-Well Plate (Cat. # EGLP-35K, Merck, Darmstadt, Germany) as per the manufacturer’s instructions. The negative control was incubated with Krebs-Ringer buffer, and the positive control with 20 mM glycylsarcosine (Gly-Sar). All analyses were performed in triplicate. The active GLP-1 stimulatory secretion effect was expressed as a percentage in relation to the negative control.

The inhibition of DPP-IV in human Caco-2 cells was determined as described previously [42]. *P. palmata* samples were rehydrated at 10 mg protein equivalent/mL in PBS buffer at 4 °C for 18 h. The solutions were centrifugated at 2580× *g* for 5 min, then sterilised by filtration using 0.2 µm syringe filters. Cytotoxicity was tested at concentrations of 1.0, 2.5 and 5.0 mg protein equivalent/mL following 1 h of treatment using PrestoBlue™. Caco-2 cells were incubated with concentrations of 1.0, 2.5 and 5.0 mg of protein equivalent/mL (final concentration).

### 2.9. Cell-Based Antioxidant Activity Assessment

The protective effect against reactive oxygen species (ROS) in human hepatic HepG-2 cells was determined after incubation with PPPE, PPPHs and the SGID samples as described previously [43]. *P. palmata* samples were rehydrated at 10 mg protein equivalent/mL in HBSS at 4 °C for 18 h. The solutions were centrifugated at 2580× *g* for 5 min, then sterilised by filtration using 0.2 µm syringe filters. Cytotoxicity was tested at concentrations of 1.0, 2.5 and 5.0 mg protein equivalent/mL following 2 h treatment using PrestoBlue™. HepG-2 cells were incubated with concentrations of 2.5 mg of protein equivalent/mL. HepG2 cells were seeded into black 96-well plates at a cell density of 8 × 10^4^ cells per well. Following 24 h incubation at 37 °C and 5% CO, cells were washed twice with PBS. 2′,7′-dichlorofluorescin di-acetate (DCFH-DA, 50 μL (25 μM final)) was added to each well. Fifty millilitres of *P. palmata* samples (final concentration: 2.5 mg of protein equivalent/mL) or N-acetylcysteine (NAC, positive protect control, final concentrations: 5 mM and 2.5 mM) was incubated with cells for 1 h. Cells were washed twice with PBS, and 100 μL ABAP (600 μM in HBSS) was added for 1 h. The plates were placed in a micro plate readerSynergy HT BioTek (Winooski, VT, USA). Fluorescence (Ex/Em 560/635 nm) was measured with excitation at 485 nm and emission at 535 nm at a sensitivity of 100.

### 2.10. Statistical Analysis

All extractions were carried out in independent triplicates. Results are expressed as mean ± standard deviation (SD). IBM SPSS version 29.0 was used for statistical analysis. Data was checked for normal distribution using the Shapiro–Wilk test and for homogeneity of variances using Levene’s test. One-way ANOVA was used to test differences among the different enzymes under the same extraction protocol with Tukey’s test used as the post hoc test. Student’s *t*-test was used to compare each hydrolysate with its corresponding SGID sample in the *in vitro* antioxidant and DPP-IV inhibitory activity assays.

## 3. Results and Discussion

### 3.1. Method Development for Protein Extraction

As extraction protocol 3 was performed at 4 °C, as it is a temperature that is used in the food industry and that may be applied during overnight processing in tanks, it was important to verify that the commercial carbohydrase preparations retained catalytic activity at this temperature. Since the activities reported by the manufacturers are generally determined under optimal assay conditions (30–65 °C), which are typically higher than those used in this study, the endo-cellulase and endo-xylanase activities at 4 °C were determined using the endo-cellulase (CellG5 method, EP 2740800 AI) and endo-xylanase (XylX6 method) assay kits, respectively, following the manufacturer’s instructions. The endo-xylanase activity at 4 °C was 91.86 ± 8.15 U/mL, 35.05 ± 2.70 U/g, 470.23 ± 7.41 U/mL, 322.65 ± 10.39 U/mL and 554.23 ± 8.46 U/g for DEPOL^TM^ 692L, DEPOL^TM^ 761P, DEPOL^TM^ 793L, CELLULASE 13L and EXTRALYVE LA2X, respectively. The endo-cellulase activity at 4 °C was 80.30 ± 1.95 U/mL, no activity was detected, 103.62 ± 0.43 U/mL, 116.15 ± 0.43 U/mL and 90.08 ± 4.43 U/g for DEPOL^TM^ 692L, DEPOL^TM^ 761P, DEPOL^TM^ 793L, CELLULASE 13L and EXTRALYVE LA2X, respectively. It was surprising to find that all the cellulase-containing preparations studied also exhibited endo-xylanase activity, whereas the xylanase preparation (DEPOL^TM^ 761P) did not exhibit endo-cellulase activity. Moreover, as the aim was to obtain intact proteins and not hydrolysates, the presence of protease activity was also tested. No endoproteinase activity was detected in any of the carbohydrase preparations as determined using the azocasein assay, as previously described [38].

#### 3.1.1. Impact of Extraction Protocol on Protein Extraction

Three different protocols were assessed for protein extraction (Figure 1). Protocol 1 was initially selected to reduce processing time and improve the potential industrial applicability of the extraction process as similar incubation periods are commonly employed for enzyme-assisted extractions. However, the protein yields obtained under these conditions were lower than those previously reported for *P. palmata* following overnight extraction at 4 °C without EAE [15]. This suggested that a shorter incubation at elevated temperature was not optimal for protein recovery. Protocol 2 was subsequently designed to combine an overnight incubation at 4 °C with additional 4 h enzymatic treatment at 50 °C, a temperature close to the optimum activity range of the commercial enzymes employed. Nevertheless, protein recovery remained lower than anticipated. Based on these findings, protocol 3 employed an overnight enzymatic treatment at 4 °C. Under these conditions, protein extraction yields were comparable to those obtained previously in the control extraction without enzyme and higher yields were achieved with carbohydrase-assisted extraction (Table 1).

The extraction method significantly affected protein recovery when different carbohydrases were used. Overall, incubation of the biomass at 50 °C at any stage of the process reduced CHOase-aided protein extraction from the *P. palmata* biomass. For example, protein extraction with DEPOL^TM^ 692L yielded only 19.37 ± 1.53 mg protein/g dw initial biomass using protocol 1, which involved incubation at 50 °C, compared with 43.08 ± 3.53 mg protein/g dw initial biomass using protocol 3, which employed incubation at 4 °C.

Heat inactivation of the enzymes at 85 °C also resulted in lower protein yields. Using protocol 3, protein extraction ranged from 22.33 ± 1.99–43.62 ± 3.73 mg protein/g dw initial biomass following heat inactivation, whereas yields ranged from 56.58 ± 7.83–79.50 ± 2.69 mg protein/g dw initial biomass in the absence of a heat-inactivation step (Table 1). Using the same protocol, EAE significantly increased protein yields from 33 to 230% compared with the control. Therefore, further studies were performed using EAE and protocol 3, omitting the heat-inactivation step.

For the first time, EAE followed by alkaline extraction of *P. palmata* proteins was evaluated at two temperatures, 4 and 50 °C. The lower temperature was chosen as a temperature routinely used in the food industry as previously indicated herein and arising from previously optimised extraction protocols [13], whereas 50 °C was chosen because it is in the region of the optimum temperature for carbohydrase activity according to the manufacturers’ specifications and reflects enzyme incubation conditions routinely used in our laboratory. Although the carbohydrase activity at 4 °C was lower than that reported in the technical documentation provided by the manufacturers, measurable activity was still detected under these conditions (see Section 2.1 and Section 2.3), indicating that incubation with the CHOases at 4 °C could still contribute to cell wall disruption and protein release.

Protein extraction results (Table 1) demonstrated that incubation at 50 °C for 4 h, as well as exposure to higher temperatures (85 °C for 10 min), reduced protein recovery irrespective of whether EAE was applied or not. These findings suggest that heat treatment, i.e., incubation at 50 °C and the enzyme heat-inactivation step, negatively affected protein extractability, potentially through protein denaturation and subsequent aggregation, resulting in a lower proportion of soluble proteins available for extraction. The major proteins in red seaweeds are phycobiliproteins, including phycoerythrin, which may account for approximately 12% of the total protein content [3,44]. Phycobiliproteins are known to be thermolabile. Previous studies have reported that R-phycoerythrin and B-phycoerythrin remain largely unaffected after incubation in PBS (pH 7.1) at 40 °C for 1 h, whereas approximately 50% denaturation occurs at 60 °C, as assessed by fluorescence and absorbance measurements [45]. Therefore, the lower protein yields in protocols 1 and 2, as well as in protocol 3, all of which included a heat-inactivation step, are likely attributable, at least in part, to thermal denaturation and reduced solubility of these abundant protein fractions. Furthermore, as no protease activity was detected in the CHOse preparations, which could otherwise continue to hydrolyse proteins to peptides during the incubation or the subsequent extraction steps, heat inactivation was not applied during protein extraction. This appears to be the first report of the impact of thermal treatments during CHOase-aided extraction or indeed during alkaline solubilisation and acid precipitation on macroalgal protein extraction yield.

#### 3.1.2. Impact of EAE on Protein Extraction, Protein Recovery Yield and Biomass Recovery Yield

**Figure 2:**
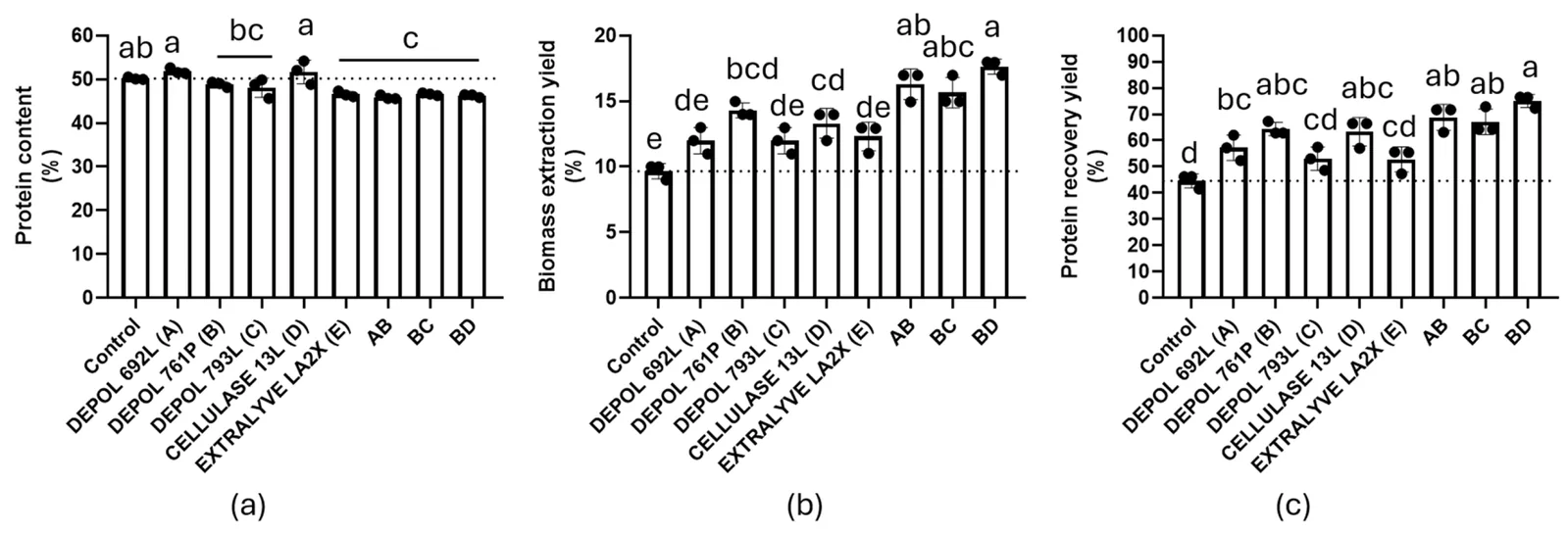
Extraction performance parameters of *Palmaria palmata* protein extracts obtained using enzyme-assisted extraction (EAE) with individual carbohydrases or a combination of carbohydrase enzymes. (**a**) Protein content (%, g of protein per 100 g of extracted biomass). (**b**) Biomass extraction yield (%, g of extracted biomass per 100 g of initial biomass). (**c**) Protein recovery yield (%, g of extracted protein per 100 g of initial protein in the biomass). Bars, error bars and dots represent average, standard deviation and individual values, respectively (n = 3). The discontinuous dotted line parallel to the x-axis indicates the average value of the control extract without EAE. Different letters denote significant differences (*p* < 0.05).

To assess extraction performance and parameters of potential industrial relevance, protocol 3 was applied at the laboratory scale using individual enzymes, combinations of cellulases (DEPOL^TM^ 692L, DEPOL^TM^ 793 L and CELLULASE 13L^®^) with the xylanase (DEPOL^TM^ 761P), and EXTRALYVE LA2X^®^, an enzymatic preparation containing both cellulase and xylanase activities (Section 2.2). Protein content, protein extraction yield, and biomass extraction yield were subsequently determined (Figure 2). The protein content ranged from 45.87 ± 0.46% (*w*/*w*) for the protein extracted with the combination of DEPOL^TM^692 L and DEPOL^TM^ 761 P to 51.82 ± 0.68% for the protein extracted with DEPOL^TM^ 692 L (Figure 2a). Statistical analysis of the protein contents obtained indicated significant differences in protein content using the individual enzymes versus a combination of enzymes (*p* < 0.01).

The biomass extraction yield ranged from 9.66 ± 0.58% in the control treatment (without EAE) to 17.64 ± 0.57% when DEPOL^TM^ 761P was combined with CELULASE 13L (Figure 2b). There were statistically significant differences in the biomass extraction yields (*p* < 0.01). Protein recovery yield, which reflects the efficacy of the extraction process by relating the amount of protein recovered to the protein content of the initial biomass (see Section 2.4), ranged from 44.64 ± 2.66% for the control treatment to 75.10 ± 2.44% for the protein extracted with the combination of DEPOL^TM^ 761P and CELULASE 13L^®^ (Figure 2c). This corresponded to a 1.68-fold increase in protein extracted relative to the control. There was a statistically significant difference in the protein recovery yield among the different CHOases employed (*p* < 0.01). Although the protein content of the extract obtained using DEPOL^TM^ 761P and CELULASE 13L^®^ was lower than that of the control, this enzyme combination was selected for further study because it provided the highest overall protein recovery and biomass recovery yields. Consequently, additional PPPE was generated using this EAE protocol with the combination of DEPOL^TM^ 761P and CELULASE 13L^®^ and subsequent hydrolysis using different proteases (Section 2.4).

The protein contents obtained herein were comparable to values previously reported in the literature [13,16]. Idowu et al. [13] investigated protein content, protein recovery yield and biomass extraction yield following carbohydrase EAE using a combination of cellulase and xylanase, followed by alkaline extraction with different alkaline solutions. The protein contents, protein recovery yields and biomass extraction yields ranged from 36.65 ± 0.89 to 56.64 ± 0.43%, 7.06 ± 0.36 to 70.65 ± 1.38%, and 3.77 ± 0.10 to 21.99 ±0.43%, respectively. Similar protein contents, protein recovery yields and biomass extraction yields were obtained herein to those reported by Idowu et al. [13] when KOH and NaOH were used as alkaline extraction agents. The reduction in protein content following EAE observed herein has also been reported by other authors [14], who attributed this effect to the solubilisation and co-extraction of non-protein components, particularly sulphated polysaccharides, during the enzymatic treatment and subsequent isoelectric precipitation [46]. Figure S2 shows the SDS-PAGE profiles of the PPPE obtained in this study. As can be observed, similar protein bands were obtained using the different CHOases, and no additional low-molecular-weight bands resulting from proteolytic degradation were detected, confirming that the CHOases did not exhibit proteolytic activity. Despite differences in extraction conditions, in this case and in the literature, it appeared to reach an upper limit in terms of protein enrichment, with maximal protein contents remaining close to 50–55% [13,16]. This suggests that EAE alkaline-based extraction approaches may have a limited capacity to further increase protein purity without the incorporation of additional downstream processing steps.

The implementation of purification technologies such as membrane filtration or ultrafiltration would likely be required in order to obtain protein ingredients with higher protein concentrations, as is the case in the dairy industry during the production of milk protein concentrates or isolates. However, increasing protein concentration is often associated with a reduction in both protein recovery and overall product yield due to the removal of non-protein solids and the loss of soluble proteins during processing. Therefore, a balance may need to be established between achieving high protein concentration and maintaining acceptable extraction and biomass yields from an industrial perspective.

The protein recovery yields obtained in this study were consistent with those reported in previous studies employing alkaline extraction, either alone or in combination with EAE [13,15,16]. In contrast, limited information is available in the literature regarding biomass yield, despite its relevance for industrial implementation. Biomass yield provides a direct indication of the amount of final ingredient that can be recovered from the initial raw material and therefore has important economic implications. The highest biomass yield obtained was 17.64 ± 0.57%. Consequently, more than 80% of the original biomass was removed during processing. From a biorefinery perspective, this residual fraction should not be considered waste but rather a potential feedstock for the recovery of other valuable compounds, such as sulphated polysaccharides, fibres, phenolic compounds or other bioactive molecules, using a cascade biorefinery concept [47]. Such an approach would contribute to improving the overall valorisation of the biomass and enhance the sustainability and economic viability of the process.

### 3.2. Physicochemical Characterisation

The final DH of the hydrolysates and their corresponding SGID samples is presented in Table 2. The extent of hydrolysis was strongly influenced by the protease employed. The highest DH was obtained with Neutrase^®^ (3.37 ± 0.05%), while the lowest DH was observed for the hydrolysate produced using Bromelain (0.44 ± 0.04%). These differences likely reflect the distinct substrate specificities, catalytic efficiencies, and cleavage patterns of the enzymes used. Following SGID, all samples showed similar DH values.

The DH values obtained herein were considerably lower than those reported by Ghelichi et al. [30], who observed DH values ranging from 43.8 to 70.7% using Alcalase^®^, Flavourzyme^®^ or Formea^®^ prime or a combination thereof. Several factors may explain these differences. First, the hydrolysis conditions differed substantially between studies. In the present work, protein hydrolysis was conducted at 50 °C for 4 h, whereas Ghelichi et al. [30] performed hydrolysis at 50 °C and incubated for 24 h. The longer incubation time would have allowed a greater number of peptide bonds to be cleaved, resulting in a substantially higher DH. In addition, differences in enzyme-to-substrate ratio (E:S) may also have contributed to the observed variation in hydrolysis efficiency; while an E:S of 1% was used in the present study, Ghelichi et al. [30] investigated E:S values of 1, 2 and 3% and the higher enzyme loadings may have promoted a higher extent of hydrolysis. Moreover, additional factors such as substrate concentration, uncontrolled (free-fall) pH conditions, enzyme preparation specificity, protein solubility, structural modifications of proteins following EAE and possible enzyme inhibition by co-extracted compounds may also account for the differences observed between studies and for the relatively low DH obtained herein.

SGID further increased the DH of all samples (Table 2), demonstrating that inclusion of the gastrointestinal enzymes continued to hydrolyse the proteins and peptides generated during the initial enzymatic treatment. This finding is consistent with the progressive breakdown of proteins that occurs during digestion and indicates that peptides not released during the hydrolysis step may become available under gastrointestinal conditions. Improvements in protein bioaccessibility following *in vitro* gastrointestinal digestion have previously been reported for PPPEs [6]. However, while these authors demonstrated enhanced protein bioaccessibility as amino acids released following digestion, they did not determine the DH of the digests, preventing a direct comparison with the present results.

**Figure 3:**
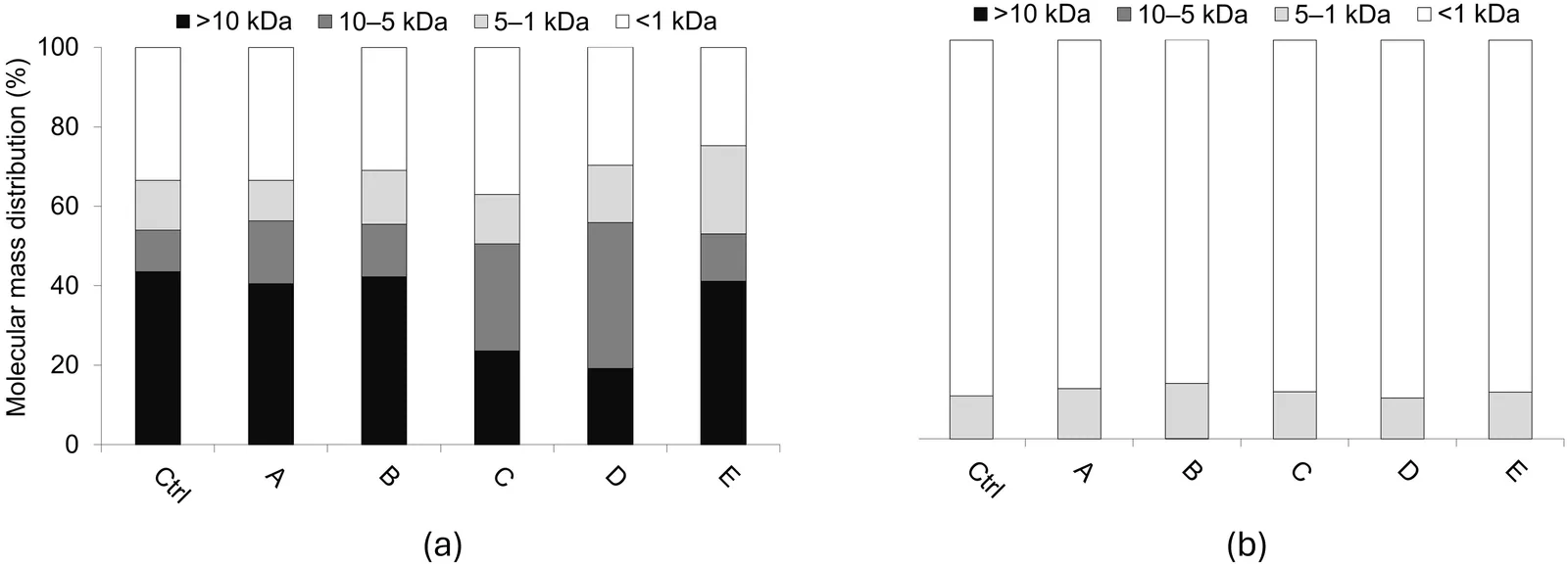
Molecular mass distribution (%) profiles of the *Palmaria palmata* protein extract obtained by enzyme-assisted extraction using DEPOL^TM^ 761P and CELULASE 13L^®^ and subsequently hydrolysed with different proteases at 50 °C for 4 h. A portion of each hydrolysate was subsequently subjected to simulated gastrointestinal digestion. (**a**) Unhydrolysed control samples and their corresponding protein hydrolysates. (**b**) Simulated gastrointestinal digest protein hydrolysates. Ctrol: unhydrolysed protein extract; A: Alcalase^®^; B: Alcalase^®^ and Flavourzyme^®^; C: Alcalase^®^ and Promod^TM^; D: Neutrase^®^; E: Bromelain, kDa: kiloDalton

Figure 3 shows the molecular mass distribution profiles of PPPE, its PPPHs and their corresponding SGID samples. Enzymatic hydrolysis decreased the proportion of high-molecular-mass components (>10 kDa and 10–5 kDa), although the extent of this reduction differed among hydrolysates. The greatest decrease in the >10 KDa fraction was observed for hydrolysates generated using the combination of Alcalase^®^ and Promod^TM^ as well as Neutrase^®^, indicating more extensive proteolysis under these conditions. Previous studies on PPPHs produced with Alcalase^®^ reported lower proportions of peptides > 10 kDa than those obtained herein [25,26]. These differences may be attributable to variations in hydrolysis conditions, including the lower substrate concentration employed by those authors (1.6% versus 10% (*w*/*v*) in the present study) and the maintenance of a constant pH of 7.0 throughout hydrolysis, both of which may have enhanced enzymatic activity and thus hydrolysis.

Simulated gastrointestinal digestion further reduced the higher-molecular-mass components of the protein fractions initially incubated with the protease preparations, resulting in samples composed predominantly of peptides ≤ 5 kDa. The molecular mass distribution profiles of all the SGID samples were quite similar. These findings are consistent with the DH values observed for the hydrolysates and SGID samples. The differences in peptide size distribution among hydrolysates before digestion are likely attributable to the distinct specificities of the proteases used. Although SGID produced similar molecular mass profiles across samples, the resulting peptide populations are likely to differ in amino acid sequence owing to differences in the peptide substrates generated during the initial hydrolysis step. Notably, peptides < 1 kDa represented the most abundant fraction following SGID, demonstrating that *P. palmata* proteins were readily digested and extensively converted into low-molecular-mass peptides.

### 3.3. In Vitro Antioxidant and DPP-IV Inhibitory Activities

The antioxidant activity of the PPPE, PPPHs and the respective SGID samples, as determined by the ORAC and ABTS assays, is presented in Table 3. Among the hydrolysates, the highest antioxidant activity was observed for the Neutrase^®^ hydrolysate using both assays, with values of 461.44 ± 37.20 μmol TE/g fdp (ORAC) and 246.48 ± 6.72 μmol TE/g fdp (ABTS). Overall, SGID increased the antioxidant activity of the *P. palmata* hydrolysates when determined using both assays.

The ORAC antioxidant activity of the unhydrolysed PPPE (121.02 ± 6.50 μmol TE/g fdp) was higher than values previously reported for aqueous extraction (39 ± 4 μmol TE/g) [48] and aqueous/alkaline extraction (81.35 ± 14.44 μmol TE/g dw) [25]. These differences may be attributed, at least in part, to differences in extraction methods, which can influence both protein recovery and the co-extraction of antioxidant compounds. In addition, seasonal variations have been reported to affect the antioxidant activity of PPPEs, with lower ORAC values in October (81.35 ± 14.44 μmol TE/g fdp) compared with April and July (210.68 ± 23.39–296.45 ± 9.18 μmol TE/g fdp) harvested biomass [26]. Therefore, the antioxidant activity observed herein falls within the range previously reported for *P. palmata*.

Significant differences in ORAC values were observed among the hydrolysates, indicating that antioxidant activity was influenced by the protease used during hydrolysis. This is consistent with previous studies showing that enzyme specificity can impact the antioxidant properties of PPPHs through the generation of peptides with different molecular characteristics and amino acid sequences [49]. Information regarding the antioxidant activity of SGID samples remains limited. However, Beaulie et al. [29] demonstrated that the <10 kDa fraction of an α-chymotrypsin hydrolysate of *P. palmata* proteins exhibited higher ORAC antioxidant activity than the corresponding >10 kDa fraction. This observation is consistent with the present results, where the SGID samples were predominantly composed of peptides < 5 kDa, with the <1 kDa fraction being the most abundant (Figure 3), and generally exhibited higher antioxidant activity than the corresponding hydrolysates prior to SGID. This suggests that the generation of low-molecular-mass peptides during gastrointestinal digestion may contribute to the enhanced antioxidant activity observed. Moreover, other co-extracted compounds, such as phenolic compounds, polysaccharides, and pigments, which may remain associated with the protein fraction during extraction, could also have contributed to the antioxidant activity observed in the ORAC assay. Liboureau and Pampanin [50] reported that enzyme-assisted extracts of *P. palmata* produced using xylanase- and cellulase-containing preparations, both containing phenolic compounds and proteins, exhibited substantial ORAC antioxidant activity. These findings suggest that, in addition to proteins and peptides, other bioactive components naturally present in *P. palmata* may contribute to the antioxidant properties of seaweed extracts.

The ABTS antioxidant activity of PPPEs and their hydrolysates has been less extensively investigated. Most previous studies have focused on ethanolic or acetonitrile extracts [51], or on isolated polysaccharide fractions [52], making direct comparisons difficult. Nevertheless, the DPPH assay, another electron transfer method, has previously demonstrated antioxidant activity in *P. palmata* extracts and their hydrolysates [30]. The ABTS results obtained herein further support the ability of *P. palmata* proteins and derived peptides to act as electron donors and exhibit antioxidant activity through electron transfer mechanisms [32], which can be relevant in food preservation and in functional foods.

Table 3 also presents the DPP-IV inhibitory activity of the samples. The Neutrase^®^ hydrolysate exhibited the strongest DPP-IV inhibitory activity, as indicated by the lowest IC_50_ value (2.11 ± 0.18 mg/mL). Furthermore, SGID increased the DPP-IV inhibitory activity of the *P. palmata* hydrolysates, suggesting the release of additional more potent bioactive peptides during gastrointestinal digestion. The IC_50_ values obtained for the unhydrolysed PPPE, its hydrolysates and the SGID samples were in agreement with those previously reported for this seaweed [25,26,36].

### 3.4. In Situ/Cell Culture Biological Activity of PPPE, PPPHs and SGID Samples

Glucose-management potential and antioxidant activity in cell-based assays were determined at concentrations ranging from 1.0 to 5.0 mg protein equivalent/mL, depending on the assay. These concentrations are within the same order of magnitude as those that may be encountered locally in the postprandial intestinal environment. Human studies have reported postprandial jejunal concentrations exceeding 140 mM amino acid equivalents following the consumption of a protein-rich meal, corresponding to approximately 15–16 g/L (15–16 mg/mL) of protein digestion products in the intestinal lumen [53]. Therefore, the concentrations evaluated in the present study are of physiological relevance. Nevertheless, the exact concentrations present in the intestinal lumen will vary depending on meal composition, protein source, degree of digestion, and gastrointestinal transit.

#### 3.4.1. Glucose-Management Potential in Cell-Based Assays

The glucose-management parameters evaluated herein involved insulin secretion by BRIN-BD11 cells, active GLP-1 secretion by STC-1 cells, and *in situ* DPP-IV inhibitory activity in Caco-2 cells.

**Figure 4:**
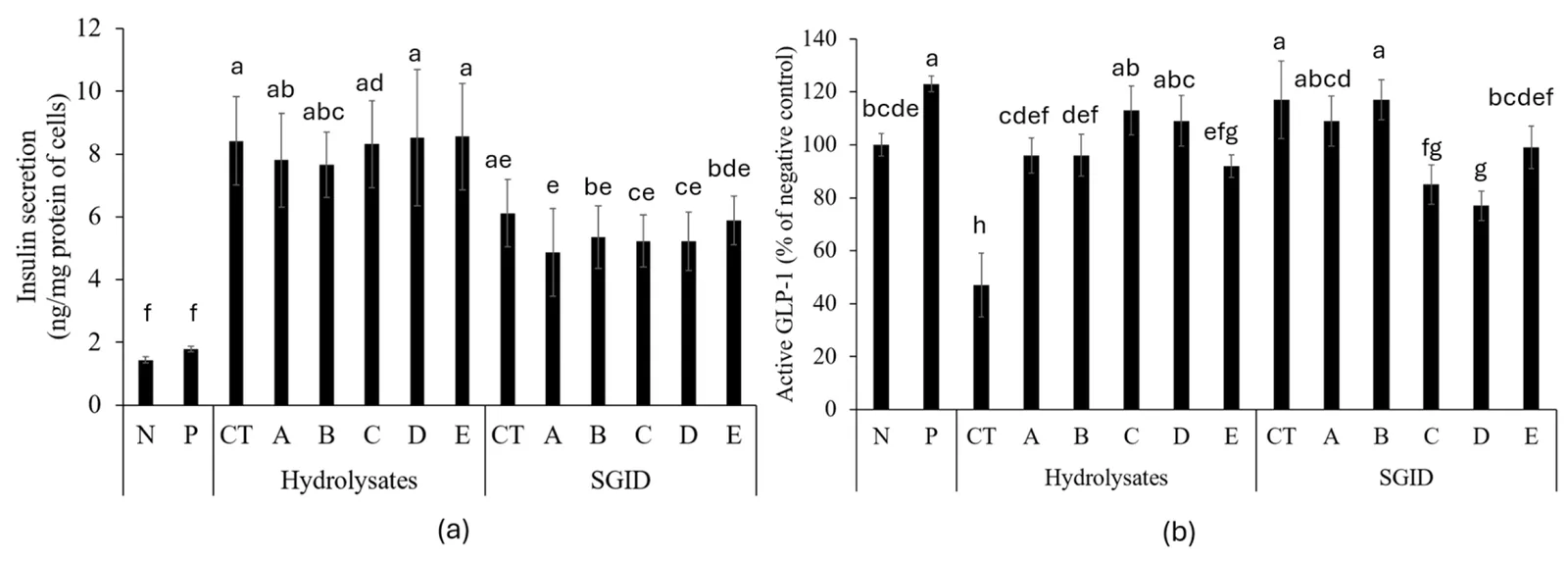
(**a**) Insulin secretion by pancreatic BRIN-BD11 cells following treatment with *Palmaria palmata* protein extract (CT), its hydrolysates, and their simulated gastrointestinal digests (SGIDs) at a concentration of 2.5 mg protein equivalent/mL for 20 min. The negative control (N) consisted of Krebs-Ringer buffer containing 1.1 mM glucose, and the positive control (P) consisted of Krebs-Ringer buffer containing 1.1 mM glucose and 10 mM L-alanine. (**b**) Active GLP-1 secretion by murine intestinal STC-1 cells following treatment with *P. palmata* protein extract, its hydrolysates, and their SGID samples at a concentration of 2.5 mg protein equivalent/mL for 4 h. The negative control (N) consisted of Krebs-Ringer buffer containing 1.8 g/L glucose, and the positive control (P) consisted of 20 mM glycylsarcosine (Gly-Sar). Bars and error bars indicate mean and SD, respectively (n = 6). Different letters denote significant differences among samples (*p* < 0.05).

PPPE, its hydrolysates or their SGID samples did not reduce BRIN BD11 or STC-1 cell viability when cells were incubated for 4 h at concentrations of 1.0 and 2.5 mg protein equivalent/mL. Insulin secretion by BRIN-BD11 cells was generally higher following treatment with the hydrolysates than with their corresponding SGID samples (Figure 4a). Among the hydrolysates, insulin secretion ranged from 7.67 ± 1.04 ng/mg cellular protein for the Alcalase^®^ hydrolysate to 8.55 ± 1.70 ng/mg cellular protein for the Bromelain hydrolysate. Although gastrointestinal digestion reduced the insulin-secretory activity of most hydrolysates, all SGID samples stimulated insulin secretion to a greater extent than both the negative and the positive control. Although the positive control (1.79 ± 0.08 ng/mg cellular protein) elicited a higher mean insulin secretion response than the negative control (1.44 ± 0.10 ng/mg protein of cells), this difference was not significant following one-way ANOVA with Tukey’s multiple-comparison test (*p* > 0.05). Therefore, the insulin-secretory activity observed should be interpreted with appropriate caution, and confirmation in additional experiments would strengthen these findings.

Active GLP-1 secretion by STC-1 cells differed among treatments (Figure 4b). Unhydrolysed PPPE exhibited the lowest stimulatory effect (47 ± 12% of negative control), whereas the SGID of PPPE (117 ± 15% of negative control) and the SGID of the Alcalase^®^ + Flavourzyme^®^ hydrolysate (117 ± 8% of negative control) significantly increased active GLP-1 secretion compared with the negative control (100 ± 4%). Prior to SGID, the Alcalase^®^ + Promod™ hydrolysate (113 ± 9% of negative control) or Neutrase^®^ hydrolyse (109 ± 10% of negative control) stimulated active GLP-1 secretion to levels comparable to the positive control (123 ± 3% of negative control). However, this effect was reduced following SGID, reaching 85 ± 7 and 77 ± 6% of the negative control, respectively. In contrast, direct simulated gastrointestinal digestion enhanced the GLP-1 secretory activity of PPPE and maintained the activity of the Alcalase^®^ and the Alcalase^®^ + Flavourzyme^®^ hydrolysates.

**Figure 5:**
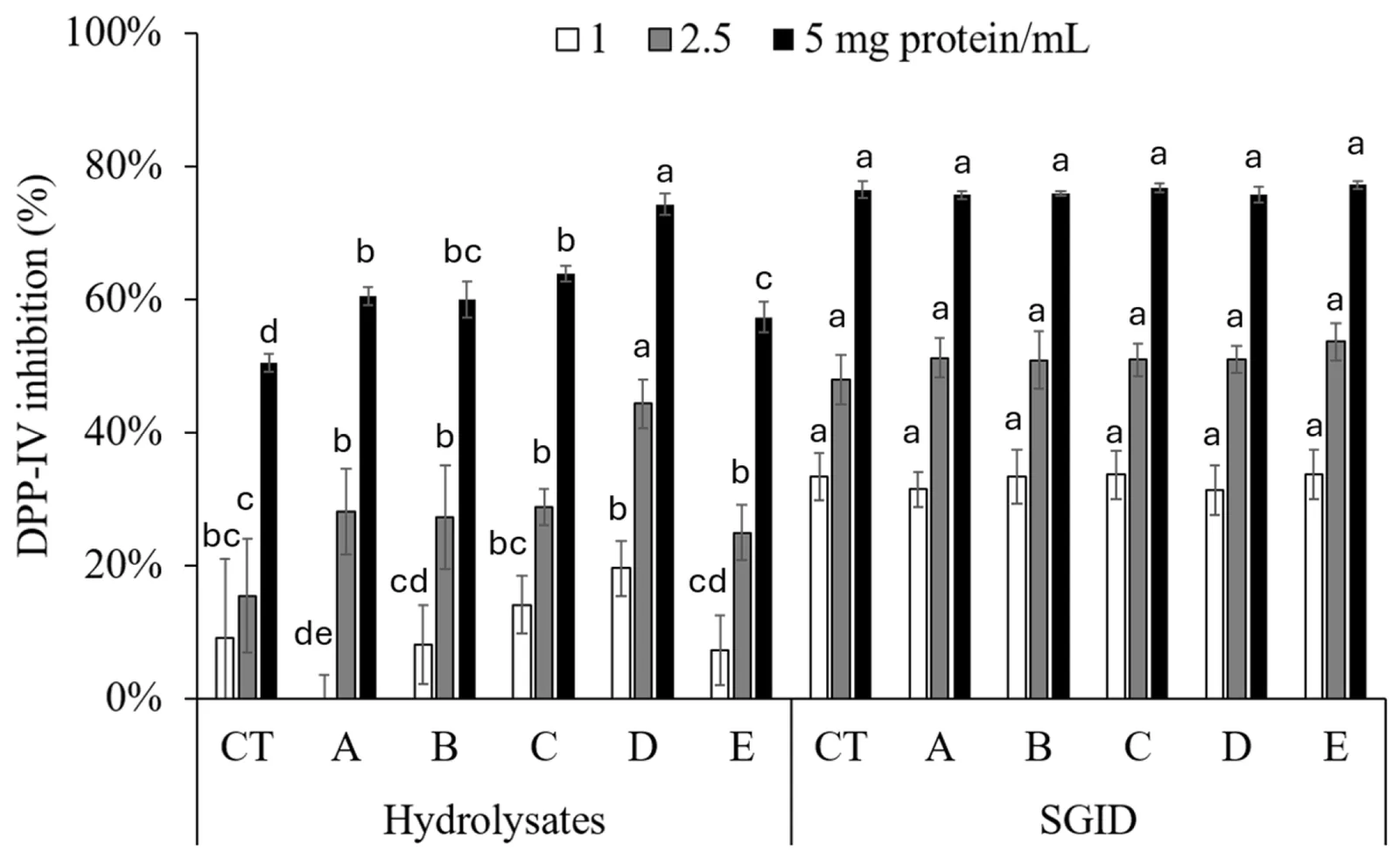
Dipeptidyl peptidase-IV (DPP-IV) inhibitory activity in Caco-2 cells treated with *Palmaria palmata* protein extract, its hydrolysates and their simulated gastrointestinal digests (SGIDs) at a concentration of 1.0, 2.5 and 5.0 mg protein/mL for 1 h. Bars and error bars indicate SE (n = 6). Different letters indicate significant differences within samples at the same protein equivalent concentration (*p* < 0.05).

PPPE, its hydrolysates or their SGID samples did not decrease Caco-2 cell viability following incubation at 1.0, 2.5 and 5.0 mg protein equivalent/mL for 1 h . All samples inhibited DPP-IV activity in Caco-2 cells (Figure 5), with inhibition generally increasing in a concentration-dependent manner. Among the hydrolysates, *in situ* DPP-IV inhibition, expressed as IC_50_, ranged from 3.12 ± 0.13 mg protein/mL for the Neutrase^®^ hydrolysate to 4.45 ± 0.18 mg protein/mL for the Bromelain hydrolysate. PPPE exhibited the weakest activity (highest IC_50_), with an IC_50_ value of 5.26 ± 0.46 mg protein equivalent/mL. Simulated gastrointestinal digestion enhanced DPP-IV inhibitory activity for all samples, resulting in IC_50_ values ranging from 2.38 ± 0.21 mg protein equivalent/mL for the Bromelain SGID sample to 2.61 ± 0.18 mg protein equivalent/mL for the Alcalase^®^ SGID sample. These findings are consistent with the *in vitro* DPP-IV inhibitory activity observed for the samples (Table 3), where SGID samples also exhibited higher inhibition than the corresponding hydrolysates.

The present findings demonstrate that PPPE, its hydrolysates and their SGID samples possess multiple bioactivities relevant to glucose homeostasis, including stimulation of insulin secretion, enhancement of GLP-1 secretion and inhibition of DPP-IV activity. These complementary results suggest that *P. palmata*-derived proteins/peptides may contribute to glycaemic management through both direct and indirect pathways. One limitation of the present study is that dose–response relationships for insulin and active GLP-1 secretion were not investigated. The primary objective was to compare the bioactivities of the different hydrolysates and their corresponding SGID samples under standardised conditions rather than to determine their potency across a range of concentrations. Therefore, future studies should evaluate a broader concentration range to establish dose–response relationships and identify the minimum effective concentrations required to elicit biological activity.

Recently, a randomised double-blind crossover human intervention study demonstrated that consumption of a beverage containing a PPPH reduced postprandial plasma glucose concentrations during an oral glucose tolerance test compared with the glucose challenge alone [54]. The present cellular findings provide mechanistic support for those observations. Previous studies in streptozotocin-induced diabetic mice reported that an Alcalase^®^ + Flavourzyme^®^ PPPH administered twice daily (50 mg/kg body weight) for 18 days increased circulating insulin concentrations while reducing blood glucose and HbA1c levels [34]. Consistent with those reports, all PPPHs evaluated herein stimulated insulin secretion from BRIN-BD11 cells. Although gastrointestinal digestion generally reduced insulin-secretory activity, the SGID samples remained more effective than both controls, suggesting that peptides generated during simulated gastrointestinal digestion retained substantial insulin secretion stimulatory activity.

The observed reduction in insulin secretion in BRIN BD11 cells following SGID treatment may be associated with further peptide hydrolysis and alterations in peptide structure during this digestion protocol. Previous work demonstrated that the insulin-secretory activity of an Alcalase^®^ + Flavourzyme^®^ PPPH decreased after simulated gastrointestinal digestion [36], indicating that some bioactive peptides responsible for stimulating insulin release may be susceptible to digestive enzyme-mediated degradation. Nevertheless, the persistence of activity after digestion suggests that some insulin-secretory peptides may be resistant to digestion and/or that additional insulin-secretory peptides are generated and may contribute to the observed response.

The effects of PPPE-derived hydrolysates on GLP-1 secretion were highly dependent on both the protease used during hydrolysis and the subsequent gastrointestinal digestion process. Interestingly, unhydrolysed PPPE displayed limited stimulatory activity, whereas a number of the hydrolysates and the SGID samples increased active GLP-1 secretion to levels similar to the positive control (Figure 4). These findings indicate that proteolysis is necessary to release peptide sequences capable of stimulating enteroendocrine cells. However, differences observed between hydrolysates before and after SGID indicate that gastrointestinal digestion may further modify peptide profiles. Depending on the peptide sequences generated during the initial hydrolysis step, gastrointestinal digestion may either degrade bioactive peptides, resulting in reduced activity, or generate new bioactive sequences with enhanced functionality. Therefore, the impact of SGID on GLP-1 secretion appears to be dependent on the characteristics of the starting hydrolysate and the susceptibility of its peptide components to further proteolysis. Previous studies have reported that PPPHs stimulate total GLP-1 secretion in GLUTag cells [36], while synthetic peptides derived from *P. palmata* protein sequences, including ILAP, LLAP and MAGVGHI, had limited activity when evaluated individually [35]. This suggests that the GLP-1 secretory effect may arise from the combined action of multiple peptides rather than from a single highly potent sequence. The enhancement observed following SGID of PPPE and the Alcalase^®^ + Flavourzyme^®^ hydrolysate further supports the generation of additional bioactive sequences during gastrointestinal digestion.

DPP-IV inhibition represents another important mechanism contributing to glucose regulation, as inhibition of this enzyme prolongs the half-life of endogenous incretins such as GLP-1 [55] and glucose-dependent insulinotropic polypeptide (GIP). The DPP-IV inhibitory activity observed both *in vitro* and *in situ* agrees with previous reports describing DPP-IV inhibitory PPPHs and peptides derived from *P. palmata* proteins [25,26,27,35,36]. Notably, simulated gastrointestinal digestion consistently enhanced DPP-IV inhibition, indicating that gastrointestinal proteolysis generates peptide populations with greater affinity for the enzyme. This observation is particularly relevant since retention of bioactivity following digestion is more representative of the peptides likely to reach the intestinal epithelium *in vivo*.

The enhanced DPP-IV inhibitory activity observed following SGID may be associated with the generation of short peptides possessing structural features that mediate DPP-IV inhibition. Previous structure–activity relationship studies have demonstrated that peptides containing proline or alanine at the penultimate N-terminal position, X-Pro motifs, hydrophobic or aromatic residues near the N-terminus, and relatively short chain lengths (particularly di- to hexapeptides) often display strong DPP-IV inhibitory activity [56,57]. Recently, Santos-Hernández et al. [58] identified several peptides with these characteristics in simulated gastrointestinal digests of *P. palmata* proteins following gastrointestinal digestion (INFOGEST), including TPGTSVA, LPYIN and LPPAA. Similar peptide sequences may have contributed to the DPP-IV inhibitory activity observed herein.

Taken together, the results indicate that PPPE-derived hydrolysates possess multifunctional glucose-regulatory properties. In addition to stimulating insulin and GLP-1 secretion and inhibiting DPP-IV activity, previous *in vitro* and *in silico* studies have demonstrated α-amylase and α-glucosidase inhibitory activities for PPPHs [30,31,33]. Therefore, these ingredients may influence glucose homeostasis through multiple complementary mechanisms, acting simultaneously at the levels of carbohydrate digestion, incretin preservation and insulin secretion. Such multifunctionality may explain the glucose-lowering effects previously observed in animal models and in human studies.

#### 3.4.2. Antioxidant Activity in HepG-2 Cells

PPPE, its hydrolysates or their SGID did not decrease HepG-2 cell viability when cells were incubated at concentrations of 1.0 and 2.5 mg/mL for 2 h.

**Figure 6:**
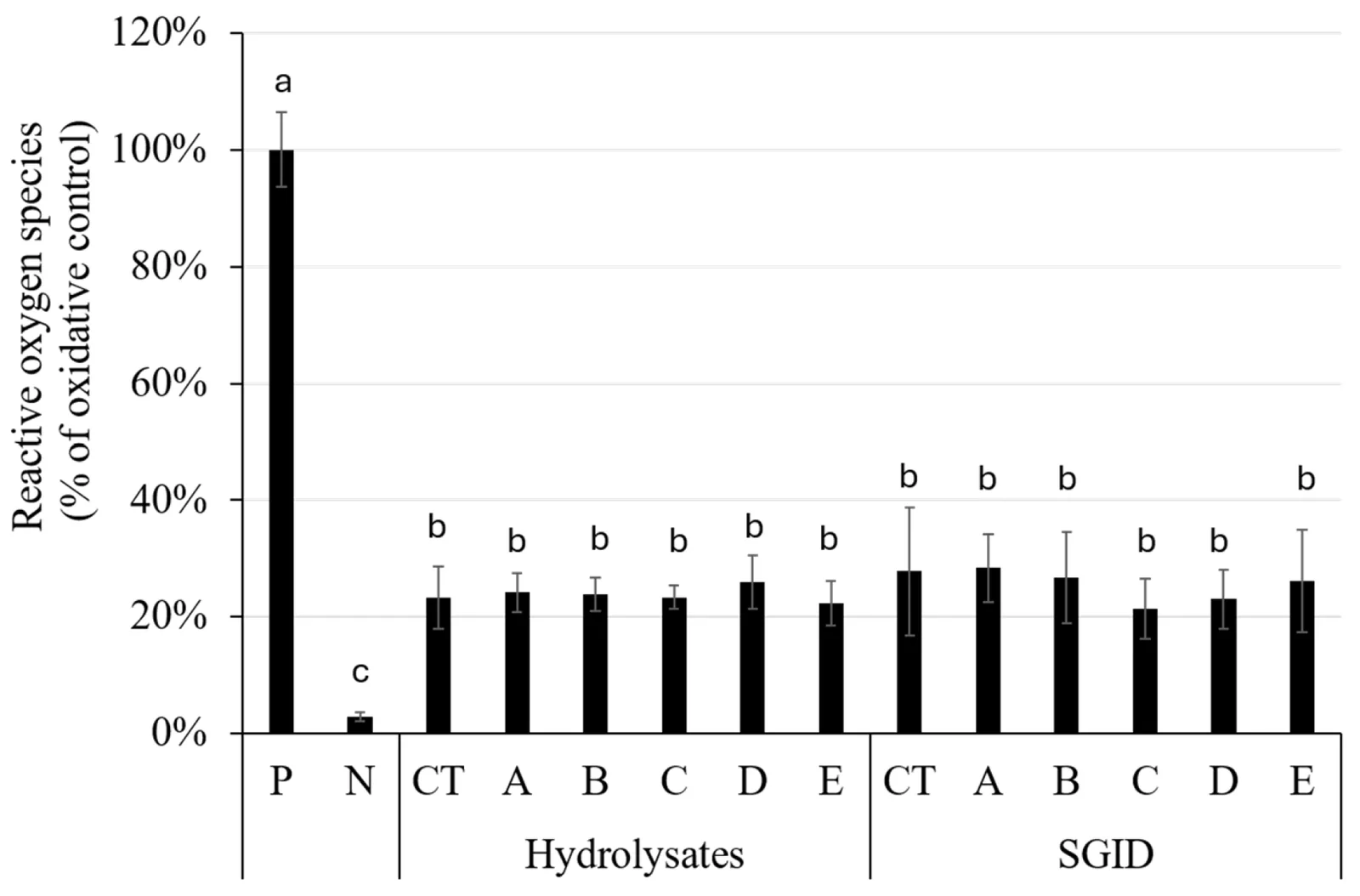
Reactive oxygen species (ROS) production in HepG-2 cells treated with *Palmaria palmata* protein extract, its hydrolysates and their simulated gastrointestinal digests (SGIDs) at a concentration of 2.5 mg protein equivalent/mL for 2 h. Negative control (N) cells were treated with HBSS only. Positive control (P) cells were treated with 2,2′-azobis(2-amidinopropane) dihydrochloride (ABAP, 600 μM in HBSS) to induce oxidative stress. Bars and error bars indicate mean and standard deviation, respectively (n = 6). Different letters denote significant differences among groups (one-way ANOVA followed by Tukey’s multiple-comparison test, *p* < 0.05). All hydrolysate and SGID samples differed significantly from the positive control (*p* < 0.0001).

PPPE, its hydrolysates or their SGID samples had a protective effect against peroxyl radicals induced by ABAP in HepG2 cells (Figure 6). Among the hydrolysates, ROS generation decreases in relation to the oxidative control, with values that ranged from 22.33 ± 3.81% of the oxidative control for the Bromelain hydrolysate to 25.93 ± 4.58% of the oxidative control for the Neutrase^®^ hydrolysate. Similar values were obtained for the SGID samples, ranging from 21.39 ± 5.11% of the oxidative control for the Alcalase^®^ + Promod™ SGID to 28.35 ± 5.87% of the oxidative control for the Alcalase^®^ SGID.

The present findings demonstrate that PPPE, its hydrolysates and their SGID samples possess antioxidant activities against peroxyl radical-induced oxidative stress in HepG-2 cells. All samples markedly reduced intracellular ROS generation compared with the oxidative control, indicating their capacity to protect cells against ABAP-induced oxidative damage. The absence of significant differences between hydrolysates and their corresponding SGID samples suggests that antioxidant activity was maintained following SGID. The retained activity may reflect the stability of antioxidant peptides generated during hydrolysis, the formation of new antioxidant peptides during gastrointestinal digestion, or a combination of both mechanisms. These results are in agreement with those reported by Libourea et al. [59], who observed antioxidant activity in Caco-2 following treatment with SGIDs of *P. palmata* and *Alaria esculenta* followed by H_2_O_2_-induced oxidative stress. Collectively, these findings indicate that bioactive peptides released from seaweed proteins can remain active under gastrointestinal conditions and exert intracellular antioxidant effects.

The antioxidant activity observed may be attributed to the presence of peptides containing amino acid residues known to contribute to radical scavenging and electron-donating activities in *P. palmata*, as has been described *in vitro* [28,29,30]. In particular, hydrophobic amino acids (A, V, L, I, P), aromatic amino acids (F, Y, W), and sulphur-containing amino acids (M and C) have been associated with antioxidant peptide activity [60,61]. Several antioxidant peptide sequences have previously been identified in *P. palmata* proteins and their hydrolysates, including peptides enriched in hydrophobic and aromatic residues such as SDITRPGGQM or ANAATIIK which may contribute to the bioactivity observed herein [28]. Furthermore, simulated gastrointestinal digestion may generate additional low-molecular-weight peptides such as LPPAA or ALLAGDPSVLE [58] with enhanced accessibility to intracellular targets, thereby contributing to ROS reduction.

Oxidative stress is recognised as a key factor in the development and progression of type 2 diabetes mellitus and its associated complications [62]. Chronic hyperglycaemia promotes excessive production of ROS through several pathways, including glucose auto-oxidation, mitochondrial dysfunction, advanced glycation end-product formation, and activation of NADPH oxidases [63]. Elevated ROS levels contribute to β-cell dysfunction, impaired insulin secretion, insulin resistance, and vascular damage [64]. Therefore, food-derived peptides capable of reducing oxidative stress may help support glucose homeostasis and reduce the risk of diabetes-related complications. In this context, the antioxidant activity observed for PPPE, its hydrolysates and their SGID samples complement their insulin and GLP-1 secretory, and DPP-IV inhibitory activities, supporting the potential of *P. palmata*-derived protein ingredients as multifunctional agents for metabolic health enhancement.

## 4. Conclusions

The findings of this study demonstrate that aqueous carbohydrase EAE performed at 4 °C using a combination of xylanase and cellulase, followed by alkaline extraction and isoelectric precipitation, provided the highest protein and biomass recovery from *P. palmata* while preserving protein integrity. Further optimisation through additional purification steps, such as ultrafiltration and diafiltration, may improve the protein content of the extracts.

Enzymatic hydrolysis of the extracted proteins using different proteases generated hydrolysates with multiple bioactivities relevant to glucose management, including insulin-secretory activity, stimulation of active GLP-1 secretion, inhibition of DPP-IV activity, and antioxidant activity against peroxyl and ABTS radicals. Simulated gastrointestinal digestion generally preserved these bioactivities and, in some cases, enhanced (e.g., DPP-IV inhibitory) activity suggesting that bioactive peptides remain available following gastrointestinal digestion.

Collectively, these results highlight the potential of *P. palmata*-derived protein hydrolysates as multifunctional ingredients for the development of functional foods targeting glycaemic management. Nevertheless, further studies are required to identify the peptide sequences responsible for the observed bioactivities and to confirm their efficacy and bioavailability *in vivo* through acute and long-term human intervention studies in both healthy and diabetic populations.

## Figures and Tables

**Table 1:** Total protein extracted (mg protein/g dry weight initial biomass) when employing enzyme-assisted extraction using different carbohydrases (with and without heat inactivation for 10 min at 85 °C) following alkaline extraction and isoelectric precipitation (see Figure 1 for further details).

Protocol	Control	DEPOL^TM^ 692L	DEPOL^TM^ 761P	DEPOL^TM^ 793L	Cellulase 13L^®^
1	9.81 ± 0.44 ^c^	19.37 ± 1.53 ^b^	20.27 ± 1.20 ^b^	18.94 ± 0.77 ^b^	27.21 ± 0.43 ^a^
2	21.01 ± 1.95 ^c^	28.07 ± 2.23 ^b^	29.64 ± 2.67 ^ab^	32.51 ± 3.00 ^ab^	35.03 ± 2.56 ^a^
3	22.33 ± 1.99 ^b^	43.08 ± 3.53 ^a^	40.20 ± 1.48 ^a^	38.67 ± 2.30 ^a^	43.62 ± 3.73 ^a^
3 ^1^	56.58 ± 7.83 ^b^	75.28 ± 1.87 ^a^	72.10 ± 2.44 ^a^	71.53 ± 6.43 ^a^	±2.69 ^a^

^1^ Protocol 3 without heat inactivation. Values represent mean ± standard deviation (*n* = 3). Different superscript letters denote significant differences within rows (the same protocol different enzyme used, *p*-value < 0.05).

**Table 2:** Degree of hydrolysis (DH, %) of *P. palmata* protein hydrolysates obtained using different proteases and their corresponding simulated gastrointestinal digest (SGID) samples. The *Palmaria palmata* protein extract obtained using enzyme-assisted extraction with DEPOL^TM^ 761P and CELULASE 13L^®^ was hydrolysed with different proteases at 50 °C for 4 h.

Enzymes	Hydrolysate Samples	SGID Samples
Unhydrolysed	0.00 ± 0.11 ^f^	31.31 ± 2.56 ^a^
Alcalase^®^	0.94 ± 0.08 ^d^	29.25 ± 1.73 ^a^
Alcalase + Flavourzyme^®^	2.64 ± 0.11 ^b^	31.47 ± 2.77 ^a^
Alcalase^®^ + Promod^TM^	2.09 ± 0.16 ^c^	33.34 ± 0.84 ^a^
Neutrase^®^	3.37 ± 0.05 ^a^	30.79 ± 2.02 ^a^
Bromelain	0.44 ± 0.04 ^e^	32.81 ± 3.46 ^a^

Values represent mean ± standard deviation (n = 3). Superscript letters denote significant differences within columns (*p*-value < 0.05).

**Table 3:** Antioxidant activity and dipeptidyl peptidase-IV inhibitory activity of *P. palmata* hydrolysates produced using different proteases and their corresponding simulated gastrointestinal digestion (SGID) samples. *P. palmata* protein extract obtained by enzyme-assisted extraction using DEPOL^TM^ 761P and CELULASE 13L^®^ was hydrolysed with different proteases at 50 °C for 4 h. A portion of each hydrolysate was subsequently subjected to SGID. ORAC, oxygen radical absorbance capacity. ABTS, 2,2′-azinobis-(3-ethylbenzothiazoline-6-sulfonic acid) radical scavenging assay. DPP-IV IC_50_, dipeptidyl peptidase-IV inhibitory activity expressed as half-maximal inhibitory concentration.

Sample	ORAC (μmol TE/g fdp)	ABTS (μmol TE/g fdp)	DPP-IV IC_50_ Value (mg/mL)
Unhydrolysed	121.02 ± 6.50 ^f^	102.25 ± 2.35 ^e^	>5
Alcalase^®^	209.51 ± 18.64 ^e^	158.38 ± 11.70 ^de^	>5
Alcalase^®^ + Flavourzyme^®^	180.29 ± 12.72 ^ef^	160.77 ± 14.73 ^de^	5.30 ± 0.12 ^g^
Alcalase^®^ + Promod^TM^	291.41 ± 10.74 ^d^	205.94 ± 5.57 ^cd^	4.24 ± 0.18 ^a^
Neutrase^®^	461.44 ± 37.20 ^c^	246.48 ± 6.72 ^c^	2.11 ± 0.18 ^f^
Bromelain	135.86 ± 14.03 ^f^	148.00 ± 3.72 ^de^	>5
SGID-control	693.33 ± 35.77 ^a$^	958.05 ± 18.33 ^ab$^	2.46 ± 0.02 ^c$^
SGID-Alcalase^®^	636.96 ± 42.73 ^ab$^	957.79 ± 22.39 ^ab$^	2.22 ± 0.04 ^ab$^
SGID-Alcalase^®^ + Flavourzyme^®^	662.87 ± 64.38 ^a$^	941.54 ± 41.38 ^ab$^	2.94 ± 0.06 ^e$^
SGID-Alcalase^®^ + Promod^TM^	572.08 ± 13.18 ^b$^	941.55 ± 75.39 ^ab$^	2.78 ± 0.07 ^de$^
SGID-Neutrase^®^	640.37 ± 32.43 ^ab$^	972.89 ± 36.50 ^a$^	2.56 ± 0.04 ^cd$^
SGID-Bromelain	641.55 ± 54.85 ^ab$^	988.32 ± 37.58 ^a$^	2.41 ± 0.04 ^bc$^

Results are expressed as mean ± standard deviation (n = 3). Different letters within columns indicate statistically significant differences (*p* < 0.05) as determined using one-way ANOVA to compare the different hydrolysates. ^$^ indicates differences between each hydrolysate and its corresponding SGID as determined using Student’s *t*-test (*p* < 0.05).

## Data Availability

The original contributions presented in this study are included in the article/Supplementary Materials. Further inquiries can be directed to the corresponding author.

## Supplementary Materials

The following supporting information can be downloaded at https://www.mdpi.com/article/10.3390/foods15203717/s1, Figure S1: Effect of pH on the recovery of proteins from *Palmaria palmata*. Proteins were extracted from the algal biomass using carbohydrase-assisted extraction with a combination of DEPOL^TM^ 761P and CELLULASE 13L^®^ followed by alkaline extraction (see Section 2.3). Protein precipitation was then performed at pH values of 2.0, 2.5, 3.0, 3.5, 4.0 and 4.5; Figure S2: Coomassie blue stained sodium dodecyl sulphate polyacrylamide gel electrophoresis (SDS-PAGE) profiles of the *Palmaria palmata* protein extracts.
