# Supplementary material for: Enzyme-Assisted Extraction and Hydrolysis of Dulse (Palmaria palmata) Proteins: Characterisation, Antioxidant Activity and Glucose-Management Potential
Source: Foods. 2026 Oct 19;15(20):3717. doi: 10.3390/foods15203717 (PMC13704318; doi:10.3390/foods15203717)
Supplement: Supplementary file 1 [file foods-15-03717-s001.zip › foods-4597389-supplementary.pdf]

Supplementary Materials

Enzyme-Assisted Extraction and Hydrolysis of Dulse (*Palmaria palmata*) Proteins: Characterisation, Antioxidant Activity and Glucose-Management Potential

Miryam Amigo-Benavent [1,2,3], Yihong Chen [1], Anthony Temitope Idowu [1] and Richard J. FitzGerald [1,2,3,*]

[1] Proteins and Peptides Research Group, Department of Biological Sciences, Faculty of Science and Engineering, University of Limerick, V94 T9PX Limerick, Ireland; miryam.amigobenavent@ul.ie (M.A.-B.)
[2] Food, Diet and Nutrition Research Group, Health Research Institute, University of Limerick, V94 T9PX Limerick, Ireland
[3] Food Science and Technology Group, BIO3 (Pharma, Science & Engineering) Pillar, Bernal Institute, University of Limerick, V94 T9PX Limerick, Ireland
* Correspondence: dick.fitzgerald@ul.ie

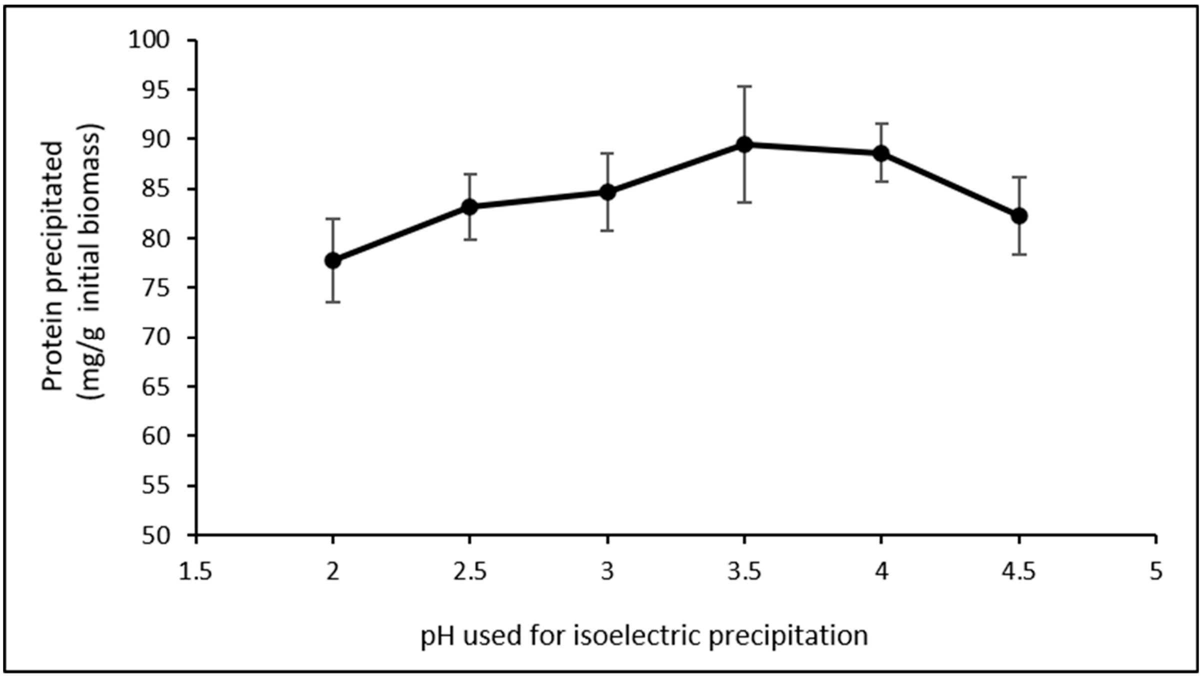

Figure S1. Effect of pH on the recovery of proteins from *Palmaria palmata*. Proteins were extracted from the algal biomass using carbohydrase assisted extraction with a combination of DEPOL^{TM} 761P and CELLULASE 13L® followed by alkaline extraction (see section 2.3). Protein precipitation was then performed at pH values of 2.0, 2.5, 3.0, 3.5, 4.0 and 4.5.

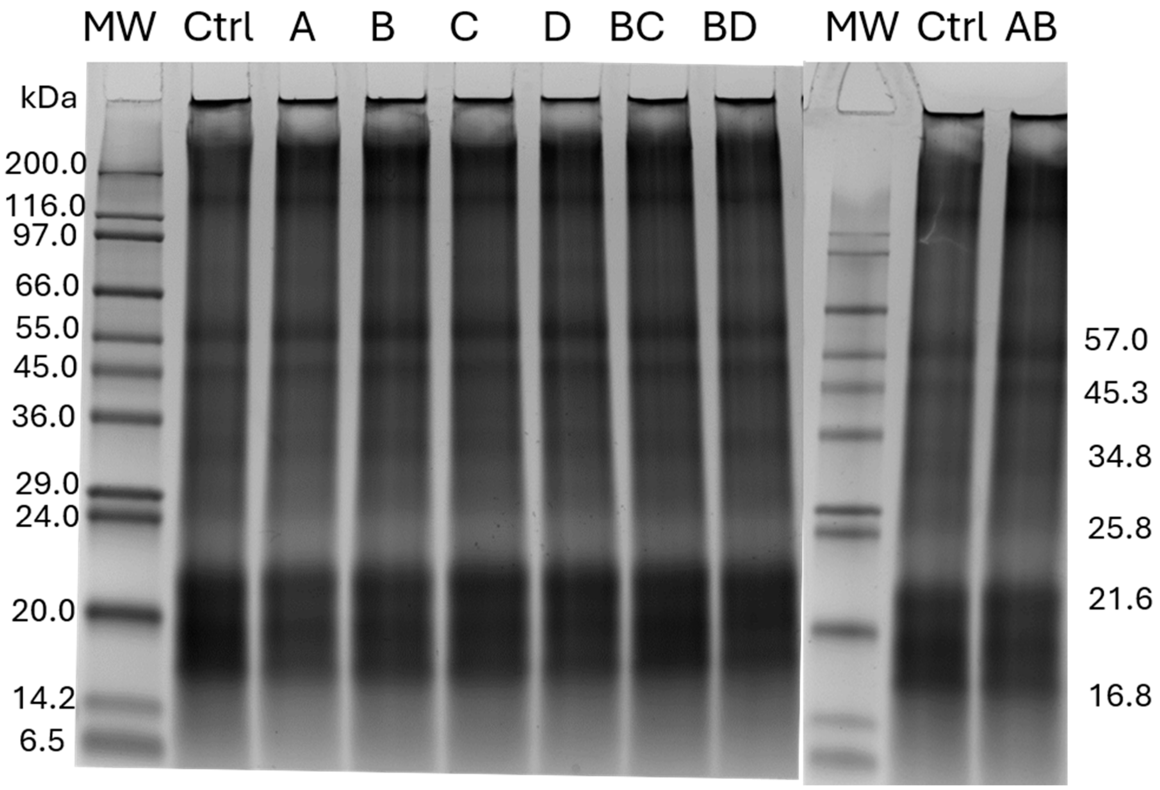

Figure S2 Coomassie blue stained sodium dodecyl sulphate polyacrylamide gel electrophoresis (SDS-PAGE) profiles of the *Palmaria palmata* protein extracts. Algal protein was extracted using individual carbohydrase (labelled A-D) or a combination of carbohydrases, followed by alkaline extraction and isoelectric precipitation at pH 3.5 as outlined in Protocol 3 (See Figure 1) without heat inactivation. All lanes were loaded with 25 ug protein/lane based on protein determination using the Kjeldhal procedure with a nitrogen to protein conversion factor of 4.7. An 8-16% precast gradient gel was used under reducing conditions. MW: molecular weight markers, Ctrl: aqueous and alkaline without enzyme-assisted extraction, where the different carbohydrases/combination of carbohydrases were as follows: DEPOL 692L (A), DEPOL 761P (B), DEPOL793L (C), CELLULASE 13L (D) or a combination of them.
